# Neutralizing Antibody Production in Asymptomatic and Mild COVID-19 Patients, in Comparison with Pneumonic COVID-19 Patients

**DOI:** 10.3390/jcm9072268

**Published:** 2020-07-17

**Authors:** Jae-Hoon Ko, Eun-Jeong Joo, Su-Jin Park, Jin Yang Baek, Won Duk Kim, Jaehwan Jee, Chul Joong Kim, Chul Jeong, Yae-Jean Kim, Hye Jin Shon, Eun-Suk Kang, Young Ki Choi, Kyong Ran Peck

**Affiliations:** 1 Division of Infectious Diseases, Department of Medicine, Samsung Medical Center, Sungkyunkwan University School of Medicine, 81 Irwon-ro, Gangnam-gu, Seoul 06351, Korea; jaehoon.ko@samsung.com; 2 Division of Infectious Diseases, Department of Internal Medicine, Kangbuk Samsung Hospital, Sungkyunkwan University School of Medicine, Seoul 03181, Korea; eunjeong.joo@samsung.com; 3 College of Medicine and Medical Research Institute, Chungbuk National University, 1 Chungdae-ro, Seowon-Gu, Cheongju 28644, Korea; krapjin@chungbuk.ac.kr; 4 Asia Pacific Foundation for Infectious Diseases (APFID), Seoul 06351, Korea; jy34.baek@gmail.com; 5 Health Promotion Center, Samsung Changwon Hospital, Changwon 51353, Korea; wonduk.kim@samsung.com; 6 Health Promotion Center, Samsung Medical Center, Seoul 06351, Korea; jaehwan.jee@samsung.com; 7 Total Healthcare Center, Kanguk Samsung Hospital, Seoul 03181, Korea; cj3.kim@samsung.com (C.J.K.); c3.jeong@samsung.com (C.J.); 8 Center for Cohort Studies, Kangbuk Samsung Hospital, Seoul 03181, Korea; 9 Division of Infectious Diseases and Immunodeficiency, Department of Pediatrics, Samsung Medical Center, Sungkyunkwan University, School of Medicine, Seoul 06351, Korea; yaejeankim@skku.edu; 10 Department of Laboratory Medicine and Genetics, Samsung Medical Center, Sungkyunkwan University School of Medicine, 81 Irwon-ro, Gangnam-gu, Seoul 06531, Korea; hyejin.shon@sbri.co.kr

**Keywords:** asymptomatic, mild, COVID-19, serology, neutralizing antibody

## Abstract

Objectives: To investigate antibody production in asymptomatic and mild COVID-19 patients. Methods: Sera from asymptomatic to severe COVID-19 patients were collected. Microneutralization (MN), fluorescence immunoassay (FIA), and enzyme-linked immunosorbent assay (ELISA) were performed. Results: A total of 70 laboratory-confirmed COVID-19 patients were evaluated, including 15 asymptomatic/anosmia, 49 mild symptomatic, and 6 pneumonia patients. The production of the neutralizing antibody was observed in 100% of pneumonia, 93.9% of mild symptomatic, and 80.0% of asymptomatic/anosmia groups. All the patients in the pneumonia group showed high MN titer (≥1:80), while 36.7% of mild symptomatic and 20.0% of asymptomatic/anosmia groups showed high titer (*p* < 0.001). Anti-SARS-CoV-2 antibodies could be more sensitively detected by FIA IgG (98.8%) and ELISA (97.6%) in overall. For the FIA IgG test, all patients in the pneumonia group exhibited a high COI value (≥15.0), while 89.8% of mild symptomatic and 73.3% of asymptomatic/anosmia groups showed a high value (*p* = 0.049). For the ELISA test, all patients in the pneumonia group showed a high optical density (OD) ratio (≥3.0), while 65.3% of mild symptomatic and 53.3% of asymptomatic/anosmia groups showed a high ratio (*p* = 0.006). Conclusions: Most asymptomatic and mild COVID-19 patients produced the neutralizing antibody, although the titers were lower than pneumonia patients. ELISA and FIA sensitively detected anti-SARS-CoV-2 antibodies.

## 1. Introduction

Over twelve million cases of coronavirus disease 2019 (COVID-19) have been reported worldwide as of 12 July 2020 [1]. In the Republic of Korea, the first COVID-19 case was reported on 19 January 2020, which was imported from Wuhan, China [2,3]. Sporadic imported and transmitted cases continued, all of which could be traced by epidemiologic investigation until the 29th patient [4]. On 18 February 2020, the 31th patient was detected and a close association with a religious group, Shincheonji, was identified. A large regional outbreak occurred around Daegu metropolitan city in association with the Shincheonji religious group, which overwhelmed the healthcare capacity of the region [4]. A rapid and massive screening system for severe acute respiratory syndrome coronavirus 2 (SARS-CoV-2) infections by reverse transcriptase polymerase chain reaction (RT-PCR) were implemented, including drive-through screening centers [5]. In addition, residential care centers designated for asymptomatic and mild COVID-19 patients were operated to alleviate the burden on hospitals using education centers of major corporations [6]. Such efforts slowed the spread of SARS-CoV-2 and led to the discovery of a large number of asymptomatic and mild COVID-19 patients [7]. The epidemiologic significance of asymptomatic and mild COVID-19 patients has been emphasized, since this population shed considerable viral load without noticeable symptoms and could be remained as undetected cases by symptom-based screening strategies [8].

Investigation of serologic responses depending on the disease severity of COVID-19 may provide essential backgrounds for practical application of serologic tests, including sero-prevalence study assessing herd immunity, sero-epidemiologic tracing of an outbreak cluster, risk assessment of healthcare workers (HCWs), and preparing convalescence plasma (CP) therapy [9,10,11,12]. To date, sero-prevalence studies have been conducted in many countries, reporting a wide range of sero-positive rates against COVID-19; 0.03% (of 3055 persons, National Health and Nutrition Examination Survey, Korea [13]), 4.06% (of 863 persons, sero-prevalence study in a community, CA, USA [9]), 4.6% (of 51,958 persons, population-based nation-wide cohort study, Spain [14]), and 23.3% (of 390 persons, donated blood products, Lombardy, Italy [15]). These different sero-positive rates might be associated with the overall size of the COVID-19 outbreak and the infection control and screening strategy of each country. For the purpose of epidemiologic tracing, there was a report from Singapore, which used serologic tests to find a missing epidemiologic link between COVID-19 clusters [5]. Such an application of serologic tests in the epidemiologic tracing would be possible in relatively less overwhelming outbreak situations. Sero-prevalence surveys were also widely conducted among HCWs and the results were quite heterogeneous according to the situations. In a sero-prevalence study among 420 HCWs who were deployed from other regions of China to Wuhan, China, the sero-positive case was zero [16]. The deployed HCWs were well educated for the use of personal protective equipment (PPE) and a stringent infection prevention and control (IPC) guideline was applied. Considering that there had been many SARS-CoV-2 infected HCWs in Wuhan during the early phase of outbreak, these data suggest that preparedness of HCWs would be essential. Other data reported various sero-positive rates of COVID-19 caring HCWs, from 0.83% (of 134 ICU HCWs, NJ, USA [17]) to 32.6% (of 285 HCWs, NY, USA [18]). These seroprevalences COVID-19 caring HCWs might be affected not only by ICP protocols, but also largely by local outbreak burdens, as sero-prevalences of non-COVID-19 caring HCWs were also high in regions of high outbreak burdens [19,20]. On the perspective of therapeutics, CP and/or hyperimmune immunoglobulin could be used, expecting an anti-viral effect of naturally produced neutralizing antibodies of recovered COVID-19 patients. For this purpose, the measurement of neutralizing antibody titers in donor plasma is essential to warrant the effect of the treatment [12,21]. However, the measurement of neutralizing antibody titers is not feasible in resource-limited settings. Data regarding the correlation between immunoassays and neutralizing activity and an understanding of the antibody response of COVID-19 patients according to the disease severity would be informative background knowledge in selecting CP donors.

Despite such a wide clinical and epidemiologic application of serologic tests against SARS-CoV-2, the understanding of serologic responses in asymptomatic and mild disease is still limited [22,23,24]. Most studies have been focused on hospitalized patients with moderate to severe illness, but the serologic response of mild patients would be different [25]. Considering that the purposes of most epidemiologic uses of serologic tests are to detect subclinical infections, an understanding of serologic response in asymptomatic and mild COVID-19 patients is essential. For this purpose, we evaluated the serologic response of asymptomatic and mild COVID-19 patients using microneutralization (MN), fluorescence immunoassay (FIA), and enzyme-linked immunosorbent assay (ELISA), in comparison with moderate to severe COVID-19 patients with pneumonia.

## 2. Methods

### 2.1. Study Population and Specimen Collection

#### 2.1.1. Asymptomatic and Mild COVID-19 Patients

We prospectively collected convalescent serum specimens from the COVID-19 patients managed at the Youngdeok Samsung residential care center. Clinically stable patients who met all of the following criteria were managed at residential care centers [6,26]; (1) alert mentality, (2) body-temperature below 37.5 °C at the diagnosis, (3) under 60 years of age, (4) no underlying disease, (5) non-smoker, and (6) no radiologic evidence of pneumonia. SARS-CoV-2 infection was confirmed by RT-PCR assay for upper and/or lower respiratory tract specimens. Patients were isolated and monitored at the center until the end of viral shedding, which was confirmed by two consecutively negative RT-PCR results. Convalescent sera after the 3rd week of illness were collected from the consented patients on the day of discharge. Information about symptom onset and duration was collected by questionnaire and telephone interview. RT-PCR test results including the cycle threshold (Ct) value were retrospectively collected. For the main analysis, patients were classified into two groups according to their symptoms; (1) the asymptomatic/anosmia group (patients without any symptoms or those only experienced anosmia and/or dysgeusia) and (2) the mild symptomatic group (patients experienced rhinorrhea, nasal stuffiness, sore throat, cough, sputum, gastrointestinal symptoms, headache, febrile sense, chilling, and/or myalgia). Patient classification with a more detailed symptom grouping is presented in the following section. Analysis data with a more detailed symptom grouping are presented in the Supplementary Materials (Tables S1 and S2 and Figure S1). The study for this population was approved by the Institutional Review Board (IRB) of Samsung Medical Center (IRB No. SMC 2020-03-120 and 2020-04-145).

#### 2.1.2. Patient Grouping of Asymptomatic and Mild COVID-19 Patients without Pneumonia

Initially, asymptomatic and mild patients were classified into four groups according to their symptoms; (1) the asymptomatic group (without any symptoms), (2) the anosmia/dysgeusia group (patients experienced anosmia and/or dysgeusia without any other symptoms), (3) the mild symptomatic group (patients experienced rhinorrhea, nasal stuffiness, sore throat, cough, sputum, gastrointestinal symptoms, and/or headache without febrile sense), and (4) the mild febrile group (patients experienced febrile sense, chilling, and/or myalgia, with or without other symptoms, although definitive fever was not documented). In addition to the pneumonia group, baseline characteristics and antibody production were evaluated according to the five severity groups (Tables S1 and S2, Figure S1).

Although the detailed characteristics and antibody production were different between the five severity groups, we decided to present the main results with larger categories of (1) the asymptomatic/anosmia group, (2) the mild symptomatic group which includes the mild febrile group, and (3) the pneumonia group, considering the following reasons. First, differentiation between asymptomatic and anosmia/dysgeusia patients were based on recalled memories to some extent. Seven of nine anosmia/dysgeusia patients were initially classified as the asymptomatic group based on the questionnaires, but recalled anosmia and/or dysgeusia symptoms on the follow up telephone interview. In addition, antibody production was quite similar between the two groups. Second, regarding the differentiation between the mild symptomatic and mild febrile groups, febrile sense would be vague and fever was not documented by a thermometer. Although FIA COI values were higher in the mild febrile group than those in the mild symptomatic group, this differentiation could be too detailed. For these reasons, we present the initial detailed grouping as Supplementary Materials for additional information, while using a more simplified grouping for the main results.

#### 2.1.3. Moderate to Severe COVID-19 Patients with Pneumonia

Serum specimens were collected from moderate to severe COVID-19 patients admitted at a tertiary care center. Clinically deteriorating or potentially severe COVID-19 patients were referred or admitted to the center. As all patients showed pneumonic infiltration on chest X-ray, these patients were classified as the pneumonia group. The patients required an oxygen supply of at least 5 L/min. Sera collected after the 1st week of illness from the consented patients were used for the main analysis. (IRB No. SMC 2020-03-113).

#### 2.1.4. Negative Control Patients

For the negative controls, sera from patients who were recovered from respiratory viral infections other than SARS-CoV-2 were collected. Infection of respiratory virus was confirmed by multiplex PCR (AdvanSure™ RV real-time RT-PCR, LG Chem, Seoul, Korea) from January to March 2020, and sera collected by the 1st week of April 2020 were used (IRB No. SMC 2020-04-125). By the time of sampling, most COVID-19 cases in Korea could be epidemiologically traced, and none of the negative control patients had epidemiologic links to COVID-19 cases or the risk area.

### 2.2. Serologic Tests for Anti-SARS-CoV-2 Antibody

#### 2.2.1. Serum Neutralization Test (MN Assay)

To evaluate the neutralization activity of the collected specimens, MN assay against SARS-CoV-2 (Korean isolate; NMC-nCoV02) was performed in duplicate using 96-well tissue culture microplates (Greiner Bio-One, Kremsmünster, Austria) in a biosafety level 3 facility. We performed a two-fold serial dilution of inactivated patients’ serum starting at a dilution of 1:10 by Dulbecco Modified Eagle Medium (DMEM) and incubated with 100 tissue culture infective dose_50_ (TCID_50)_ of the virus for 1 h at 37 °C and then infected to Vero cells. After 60 min incubation, the mixture of serum and virus was removed and DMEM media were added to the infected cells. The cells were incubated at 37 °C in 5% CO_2_ for 4 days. The supernatants were removed, fixed with 10% formalin solution, and stained with crystal violet to determine the titer. Antibody titers were defined as the highest serum dilution that inhibited the cytopathic effect (CPE), and 1:10 dilution was considered as the lowest possible significant titer.

#### 2.2.2. IgM and IgG Antibody Test (FIA Kit)

For the assessment of IgM and IgG response, an automated fluorescent lateral flow immunoassay kit (AFIAS COVID-19 Ab assay, Boditech Med Inc., Chuncheon, Korea) was used. The kit detects the anti-SARS-CoV-2 IgM and IgG antibodies using the AFIAS-6 analyzer system and disposable AFIAS cartridge [27]. This assay uses sandwich immunoassay with detector SARS-CoV-2 protein (recombinant nucleocapsid proteins with europium chelate) in a buffer and captures mAbs (Mouse anti-human IgM and anti-human IgG monoclonal antibody) immobilized on the nitrocellulose membrane in the AFIAS cartridge. One hundred microliters of blood specimen is required for the test. The specimen is mixed with detector buffer and loaded onto the nitrocellulose membrane in the cartridge. If the anti-SARS-CoV-2 nucleocapsid proteins IgM and IgG are present in the specimen, they react with the europium-labeled detector antigen to form an antigen–antibody complex and then captured by capture antibodies on the nitrocellulose membrane. After a reaction time of 10 min, the AFIAS-6 scanner measures the time resolved fluorescence intensity, expressed as a relative COI (cut-off index) value. The COI values are approximately proportional to the concentration of the IgM and IgG antibodies in the specimen. Specimens with a COI value ≥ 1.1 were considered positive. All procedures were performed according to the manufacturer’s instructions.

#### 2.2.3. Total Antibody Test (ELISA Kit)

To evaluate total antibody response, an ELISA kit (PCL COVID-19 Total Ab EIA test, PCL Inc., Seoul, Korea) was used. The ELISA kit detects the total antibodies against the nucleocapsid protein and receptor binding domain (RBD) of the spike protein of SARS-CoV-2, using a sandwich immunoassay method. The plate for ELISA is coated with the SARS-CoV-2 antigen, specifically, nucleocapsid protein expressed by *Escherichia coli* (*E. coli)* and the RBD of the spike protein expressed by HEK293S cells. After applying a positive serum or plasma to each well of the antigen coated plates, anti-SARS-CoV-2 antibodies combine with SARS-CoV-2 antigens on the surface, and unbound or non-specific bindings are washed afterward. Horseradish peroxidase (HRP)-conjugated secondary SARS-CoV-2 antigen is added for the 2nd reaction. After washing the unbound HRP conjugated, chromogen solution is added. When SARS-CoV-2 antigen–antibody–antigen (antigen sandwiched complex) is formed, HRP reacts with chromogen solution and the color changes from colorless to blue. It changes to yellow when sulfuric acid is added as a stop solution. The intensity of the color is proportional to the amount of antibodies in the specimen. All tests were performed in duplicate, according to the manufacturer’s instructions and optical density (OD) ratios ≥ 1.0 were considered positive.

### 2.3. Statistical Analysis

To compare variables between groups, either the one-way analysis of variance or the Kruskal–Wallis test was used for continuous variables, and the Chi-square test for categorical variables. All *P*-values were two-tailed, and those < 0.05 were considered statistically significant. IBM SPSS Statistics version 20.0 (IBM, Armonk, NY, USA) was used for statistical analyses.

## 3. Results

### 3.1. Clinical Characteristics of the COVID-19 Patients

Overall, 70 laboratory-confirmed COVID-19 patients were enrolled, including 15 asymptomatic/anosmia, 49 mild symptomatic, and 6 pneumonia patients (Table 1). For the patients managed at the residential care center (asymptomatic/anosmia and mild symptomatic groups), one serum specimen was collected per patient on the day of discharge (median 36 days from diagnosis), while 18 serum specimens were obtained from the pneumonia group on different days after the 1st week of illness (median 19.5 days from diagnosis). As the negative controls, 21 serum specimens were collected from patients recovered from other respiratory virus infections (Table S3). Patients in the asymptomatic/anosmia and mild symptomatic groups were significantly younger than in the pneumonia group (mean age 25.2, 30.9, and 65.7 years, respectively; *p* < 0.001). Male to female ratios were around 1:1 and not statistically different between groups. Patients in the asymptomatic/anosmia and mild symptomatic groups did not have underlying diseases, while all the patient in the pneumonia group had diabetes mellitus and some additional underlying diseases (presented as a footnote of Table 1). Overall, the symptom duration of rhinorrhea and/or nasal stuffiness was the longest (median 21 days, Interquartile range (IQR)10.5–50.5), followed by anosmia/dysgeusia (median 14 days, IQR 7.5–24.0). Mild symptoms were not accurately assessed in patients of the pneumonia group, as they were severely ill during the initial course of the disease, including two patients with mechanical ventilation support and one with a high flow nasal cannula. All patients in the pneumonia group experienced fever which persisted for 15 days in median. Peak viral loads were significantly higher in the pneumonia group, with the lowest Ct value of 26.4 in median (*p* = 0.003). The duration of viral shedding was not statistically different between groups, although the median value of duration was numerically shorter in the pneumonia group (23 for pneumonia and 32 for other groups, days from diagnosis).

### 3.2. Antibody Production of Asymptomatic to Pneumonic COVID-19 Patients

**Figure 1:**
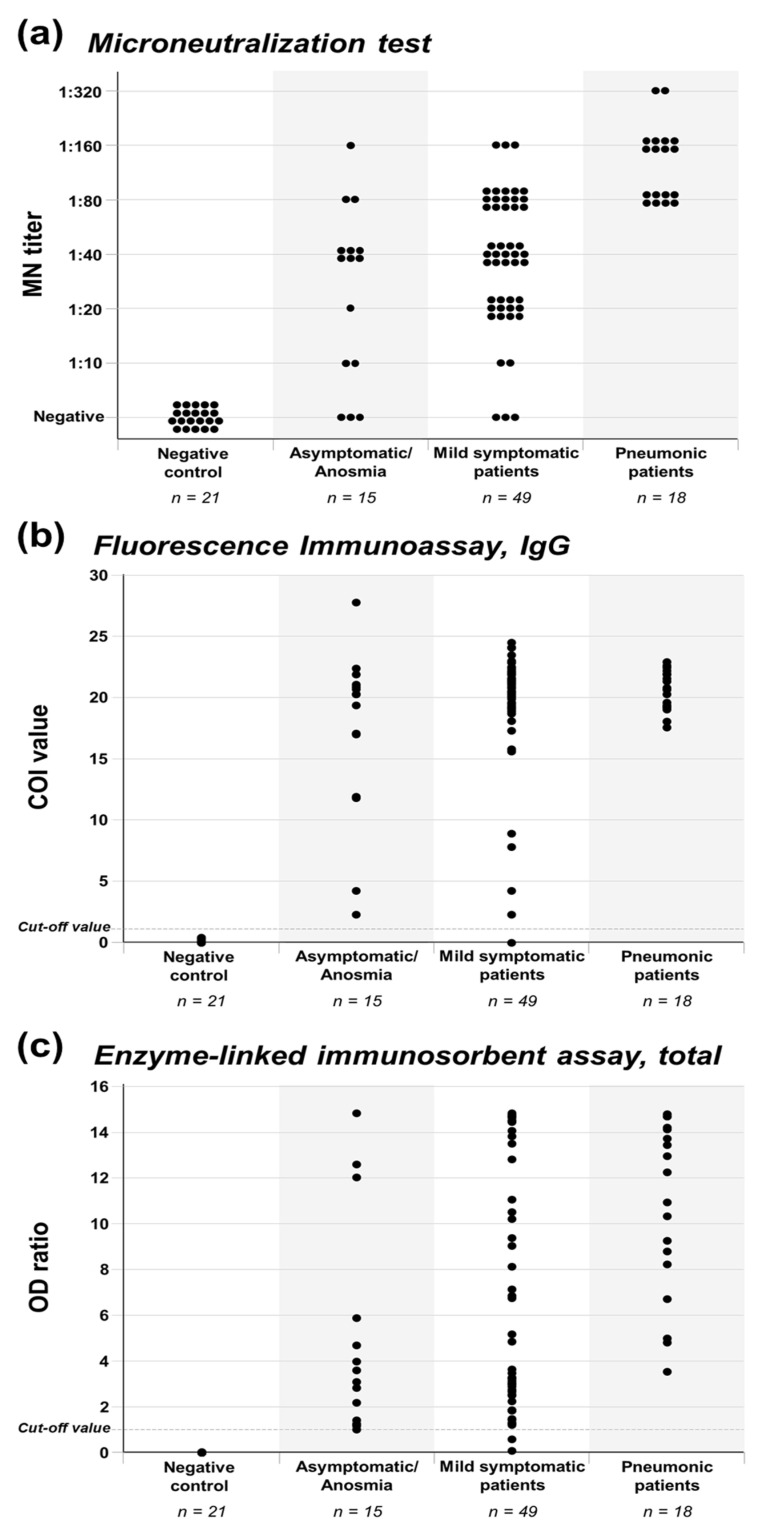
Antibody production of asymptomatic/anosmia, mild symptomatic, and pneumonic COVID-19 patients. Antibody production of asymptomatic/anosmia, mild symptomatic, and pneumonic COVID-19 patients was evaluated by (**A**) MN, (**B**) FIA IgG, and (**C**) ELISA methods.

Antibody production of asymptomatic to pneumonic COVID-19 patients are presented in Figure 1 and Table 2. The neutralizing antibody was detected in 91.4% (64/70) of the enrolled COVID-19 patients. All the patients in the pneumonia group produced the neutralizing antibody (100%). Production of the neutralizing antibody was observed in 93.9% of the mild symptomatic group and 80.0% of the asymptomatic/anosmia group. Although the proportion of neutralizing antibody production was lower in the milder groups, statistical significance was not noticed (*p* = 0.079). In the comparison of high MN titers between groups, all the patients in the pneumonia group produced the neutralizing antibody with MN titer ≥ 1:80, while 36.7% of the mild symptomatic group and 20.0% of the asymptomatic/anosmia group showed MN titer ≥ 1:80 (*p* < 0.001). Different proportions of high MN titers between severity groups and statistical significance were also noticed in the comparison of the five severity groups (*p* < 0.001, Table S2).

With the FIA and ELISA methods, the antibody against SARS-CoV-2 in the asymptomatic/anosmia and mild symptomatic groups could be detected with a higher proportion than the MN test. All the patients in the asymptomatic/anosmia group were positive for FIA IgG and ELISA. Only one or two patients in the mild symptomatic group were negative for FIA IgG and ELISA, respectively. For the FIA IgG test, all patients in the pneumonia group exhibited a COI value ≥ 15.0, while 89.8% of the mild symptomatic group and 73.3% of the asymptomatic/anosmia group showed a COI value ≥ 15.0 (*p* = 0.049). For the ELISA test, all patients in the pneumonia group showed an OD ratio ≥ 3.0, while 65.3% of the mild symptomatic group and 53.3% of the asymptomatic/anosmia group showed an OD ratio ≥ 3.0 (*p* = 0.006). Different proportions of high FIA IgG COI values and ELISA OD ratios between severity groups and statistical significance were also noticed in the comparison of the five severity groups (*p* = 0.039 and *p* = 0.034, respectively; Table S2). Based on the test results of the present analysis, sensitivity was 98.8% for FIA IgG and 97.6% for ELISA. Although a false positive test result was not observed, the number of negative control samples was limited to calculate specificity.

The FIA IgM antibody was positive in three sera of the asymptomatic/anosmia and mild symptomatic patients (3/64, 4.7%), while nine sera (9/18 sera, 50.0%) from three pneumonic patients (3/6 patients, 50.0%) were positive for IgM (Figure S2). Since the positive proportions of FIA IgM were relatively low, we additionally evaluated the serial serum specimens of two COVID-19 patients with pneumonia (Figure S3). Patient A was a 46-year-old male with underlying diabetes mellitus. He was admitted on day 4 of illness and all antibody tests were negative by day 5. On day 9 of illness, all IgM, IgG, and total antibodies converted to be positive. The COI value of FIA IgM increased until discharge (day 19 of illness), while the COI value of IFA IgG and OD ratio of ELISA total antibody tests peaked on day 9 and did not show a further increment on the follow up specimens. Patient A developed pneumonia after admission and was treated with hydroxychloroquine, lopinavir/ritonavir, and inhaled ciclesonide. He required oxygen support up to 5 L/min via nasal prolong. He could fully recover from illness without mechanical ventilator support and was discharged on day 20 of illness. Patient B was a 66-year-old female with underlying diabetes mellitus. She was referred to our center on day 20 of illness due to pneumonia progression and received endotracheal intubation with mechanical ventilator support. All the antibody tests were positive from the day of referral, but IFA IgM COI values were much lower than those of Patient A measured on similar illness days. She was treated with hydroxychloroquine, lopinavir/ritonavir, and intravenous corticosteroid. Her FIA IgM COI values showed a waning trend during illness, FIA IgG COI values remained high throughout the hospitalization, and OD ratio of ELISA total antibody tests showed an increasing trend. Patient B fully recovered from the illness and was discharged on day 57 of illness. Since not all serial specimens of these patients underwent an MN test, the titers of MN titers were not presented in the figure. The MN titers of Patient A increased from day 9 of illness, and peaked on day 13 of illness (1:320). Patient B showed similar MN titers during hospitalization (1:80 or 1:160).

## 4. Discussion

In the present serologic investigation, a neutralizing antibody was detected in mild COVID-19 patients, even in asymptomatic cases. However, not all the asymptomatic and mild COVID-19 patients could produce neutralizing antibodies, and high titer production was significantly lower in asymptomatic to mild patients, compared to that of moderate to severe patients with pneumonia. Immunoassays including FIA and ELISA methods could detect past SARS-CoV-2 infections sensitively, and different productions of high FIA COI values and high ELISA OD ratios were also noticed among the severity groups. These findings may have important implications regarding the practical application of serologic tests as follows.

To cope with the current COVID-19 pandemic, a sero-prevalence investigation of a community is conducted to assess the proportion of subclinical infections and status for herd immunity [9,28,29]. This approach has three major assumptions. First, there would be a certain proportion of subclinical infections which may not be detected by a symptom-based, RT-PCR screening strategy. Subclinical cases have been reported to be 20–40%, though the undetected proportion would vary depending on the outbreak situation [8,30]. Second, subclinical infections may produce detectable antibodies against SARS-CoV-2. For the case of the Middle East respiratory syndrome coronavirus (MERS-CoV), this assumption may not be applicable since none of the asymptomatic cases and 60% of the febrile patients without pneumonia produced detectable antibodies [25]. In the present investigation, it was noticed that asymptomatic and mild COVID-19 patients could produce a detectable level of antibodies against SARS-CoV-2. One completely asymptomatic patient even showed high MN titer of 1:80, similar with pneumonic COVID-19 patients. These findings imply that asymptomatic COVID-19 patients are not likely to be at the simple carrier status. Without provoking recognizable symptoms, immunologic interactions between SARS-CoV-2 and the human body might occur silently. The immunologic phenomenon in asymptomatic and mild symptomatic COVID-19 patients needs to be further evaluated. Third, the produced antibody may protect the host from re-infection of SARS-CoV-2. However, the production of the neutralizing antibody in asymptomatic to mild febrile COVID-19 patients was apparently lower than patients with pneumonia. Although immunoassay methods including FIA and ELISA could sensitively detect anti-SARS-CoV-2 antibodies, this detection did not necessarily mean neutralization activity, especially in mild cases. It is not clear whether neutralizing antibodies with low titers may protect hosts from re-infection of SARS-CoV 2 and how long these antibodies may persist. For the proper assessment of herd-immunity and the establishment of an outbreak control strategy, further studies should be conducted for the protective capacity and persistence of neutralizing antibodies.

From the perspective of the sero-diagnosis of SARS-CoV-2 infection, there could be several clinical applications. For the epidemiologic investigation of an outbreak cluster, serologic studies can be used to detect the asymptomatic index case of a cluster [5]. The finding of the present study is that most asymptomatic and mild COVID-19 cases produced detectable antibodies which supports the application of serologic tests for epidemiologic tracing. Both immunoassay methods of FIA IgG and the ELISA total antibody test showed a high sensitivity in detecting past SARS-CoV-2 infection regardless of the severity of illness. Considering the mechanism of tests, other immunoassay methods with well validated performances could be used in sero-epidemiologic studies. Meanwhile, based on the findings of the present analysis, negative neutralizing tests cannot exclude past infections since 20% of RT-PCR confirmed asymptomatic/anomia COVID-19 patients were negative for neutralizing tests. Although we did not calculate the specificity of the IFA and ELISA kits due to the limited number of negative control specimens, it was noticed that a cross-reactivity to convalescence sera from other respiratory virus infections was not observed. This finding has a similar implication for the serologic evaluation for healthcare workers (HCWs) who are caring for COVID-19 patients. Due to the high infectivity of SARS-CoV-2 and the heavy work burden to HCWs during an outbreak situation, HCWs are considered a high-risk group for SARS-CoV-2 infection. By detecting past asymptomatic infections of HCWs, the overall risk and protective measures for HCWs can be evaluated. Sero-prevalence studies published to date are presented in the introduction section. Whether to confirm positive immunoassay tests by neutralization tests, and whether HCWs with neutralizing antibodies would be safe during additional SARS-CoV-2 exposure, are still unresolved issues and need further investigations.

Serology data for various clinical spectrums of COVID-19 patients would be background knowledge for preparing CP therapy. Clinical studies in patients with acute respiratory distress syndrome caused by SARS-CoV-2 have shown a therapeutic benefit from the use of CP [12,31]. However, during the MERS outbreak in South Korea, CP was donated from relatively mild MERS patients who recovered shortly, but half of the donated plasma did not have neutralization activity [21]. Likewise, young and previously healthy COVID-19 patients are more likely to be in good general condition after illness and willing to donate CP, but they might not have a sufficient concentration of the neutralizing antibody. Instead, pneumonic patients with moderate illness may be better candidates for CP donors, since they show comparable neutralization titers to patients with severe illness with the minimum MN titer of 1:80. Although the number of pneumonic patients was limited in the present analysis, the previous serologic study by Perera et al. also exhibited that the production of the neutralizing antibody was favorable in pneumonic patients with moderate illness [24]. As measurement of the neutralizing antibody titer and preservation of collected blood product are hurdles for CP treatment, hyperimmune immunoglobulin products could be prepared to ensure the amount of neutralizing antibodies and facilitate stable supply, storage, and administration. The effect and safety of CP and hyperimmune immunoglobulin are still controversial and need further investigations [32].

The sero-kinetic evaluation of the two COVID-19 patients with pneumonia suggests several implications about the serologic test method we used. First, the sensitivity of IgG and total ELISA were quite sensitive, but the COI values and OD ratios peaked at the very early phase, which might be due to saturation of signals. Meanwhile, FIA IgM showed lower COI values compared to IFA IgG, but a linearity during the course of illness was observed in Patient A. This suggests that sensitive immune assay methods could be saturated early, and titration would be required for the evaluation of sero-kinetics of COVID-19 patients [33]. Second, based on the present study’s findings and previous reports, IgM might play a limited role in terms of serologic diagnosis. IgG and IgM converted to be positive at a similar time point in Patient A, though sera between day 5 and 9 were not available. In the serial evaluation of Patient B, the FIA IgM COI values were lower than those of IgG and showed a waning trend before the end of viral shedding. In previous reports, IgM antibodies were detected two days earlier than IgG antibodies, and IgM antibodies waned earlier than IgG antibodies [23,34]. The window period that IgM appears earlier than IgG would be very narrow, and may not even exist in mild patients in whom IgM response weakly occurs [34]. In addition, IgM antibodies in convalescent sera of mild patients were limitedly detected in the present analysis, which is compatible with other published reports [34]. Third, on the other hand, severe patients may show a suppressed antibody response. Compared to Patient A, who recovered from pneumonia without mechanical ventilator support, Patient B showed lower IgM COI values and MN titer. Although each sero-kinetics study did not include a sufficient number of patients according to detailed severities, decreased antibody response in critical COVID-19 patients was also noticed in previous reports [24].

We note that our study has several limitations. First, patient numbers of asymptomatic/anosmia and pneumonia groups were relatively limited according to the disease severity classification of the present study. However, we noticed that antibody production occurred even in asymptomatic patients, while high titer production was limited in mild groups. Second, there could be a recall bias regarding the symptoms of the patient since they were recorded after recovery from the disease. Nevertheless, initially negligible symptoms were additionally identified by telephone interview. About a half of patients who were initially classified as asymptomatic were not truly asymptomatic and recalled mild symptoms in the telephone interview, especially anosmia. Lastly, IgM antibodies were limitedly measured in the present investigation. Since we only use a single test method for IgM and did not evaluate serial serum specimens from mild patients, it is not clear whether IgM antibodies were scarcely formulated in mild disease, diminished early, or sensitivity of FIA for IgM antibodies was low. However, as discussed above, it is more likely that IgM antibody production is limited in mild COIVD-19 patients considering other reported studies. This could be further evaluated by a sero-kinetics study for asymptomatic and mild COVID-19 patients.

## 5. Conclusions

The neutralizing antibody was detected in most of the mild COVID-19 patients after the 3rd week of illness even in asymptomatic cases, although the titers were lower compared to those in pneumonia patients, and its significance needs to be elucidated. Both ELISA and FIA showed comparable sensitivities for the detection of anti-SARS-CoV-2 antibodies in patients with variable clinical manifestation, including asymptomatic COVID-19 patients.

## Figures and Tables

**Table 1:** Clinical characteristics of asymptomatic to severe coronavirus disease 2019 (COVID-19) patients.

	Asymptomatic/ Anosmia Group n = 15	Mild Symptomatic Group n = 49	Pneumonia Group n = 18 *	*p* Value
Age	25.2 ± 8.0	30.9 ± 9.0	65.7 ± 13.2	<0.001
Male sex	8 (53.3%)	20 (40.8%)	3 (50%)	0.665
Underlying disease	0 (0%)	0 (0%)	6 (100%) ^†^	<0.001
Symptom, duration				
Anomia and/or dysgeusia	9 (60.0%), 14.0 (5.5–21.0)	16 (32.7%), 18.5 (8.3–29.3)	0 (0%), NA	0.025
Rhinorrhea and/or nasal stuffiness	0 (0%), NA	17 (34.7%), 21.0 (10.5–50.5)	0 (0%), NA	0.008
Sore throat	0 (0%), NA	18 (36.7%), 6.0 (3.0–18.5)	1 (16.7%), 5.0 (NA–NA)	0.017
Cough	0 (0%), NA	25 (51.0%), 11.0 (4.5–30.0)	4 (66.7%), 10.5 (4.0–17.8)	0.001
Sputum	0 (0%), NA	17 (34.7%), 10.0 (7.0–28.0)	1 (16.7%), 14.0 (NA–NA)	0.023
Gastrointestinal symptoms	0 (0%), NA	10 (20.4%), 4.0 (2.8–7.0)	3 (50.0%), 7.0 (5–NA)	0.024
Headache	0 (0%), NA	9 (18.4%), 3.0 (1.0–5.0)	3 (50.0%), 17 (5–NA)	0.021
Fever, chill, and/or myalgia	0 (0%), NA	19 (38.8%), 3.0 (1.0–5.0)	6 (100%), 15.0 (7.3–20.8)	<0.001
Lowest Ct value	33.6 (32.4–35.7)	32.2 (30.8–34.1)	26.4 (19.3–31.1)	0.003
Duration of shedding				
from symptom onset	34.0 (29.5–41.5) ^‡^	40.0 (33.5–45.0)	29.0 (23.0–38.8)	
from diagnosis	32.0 (26.0–34.0)	32.0 (29.0–43.5)	23.0 (18.3–37.8)	0.181
Sampling time, days				
from symptom onset	39.0 (34.5–46.5) ^‡^	43.0 (37.0–49.5)	24.0 (19.8–38.3)	
from diagnosis	36.0 (30.0–38.0)	36.0 (31.5–47.5)	19.5 (16.8–35.5)	0.001

Data are expressed as the number (%) of patients, mean ± standard deviation, or median (interquartile range). * A total of 18 serum samples were obtained from six pneumonic patients. Two patients with pneumonia underwent endotracheal intubation and symptoms could not be properly assessed. ^†^ Six (100%) patients had diabetes mellitus, two (33.3%) had hypertension, one (16.7%) had dyslipidemia, one (16.7%) had stable angina, one (16.7%) had gout, and one (16.7%) had thyroid cancer. ^‡^ Values for 9 anosmia/dysgeusia patients. Abbreviations: COVID-19, coronavirus disease 2019; NA, not applicable; Ct, cycle threshold of RT-PCR.

**Table 2:** Neutralizing antibody production and serologic tests for asymptomatic to severe COVID-19 patients.

	Asymptomatic/ Anosmia Group n = 15	Mild Symptomatic Group n = 49	Pneumonia Group n = 18 *	*p* Value
Neutralizing Antibody				
Positive, titer ≥1:10	12 (80.0%)	46 (93.9%)	18 (100%)	0.079
High MN titer, ≥1:80	3 (20.0%)	18 (36.7%)	18 (100%)	<0.001
FIA, IgG				
Positive, COI ≥1.1	15 (100%)	48 (98.0%)	18 (100%)	0.711
High COI value, ≥15.0	11 (73.3%)	44 (89.8%)	18 (100%)	0.049
ELISA, Total				
Positive, OD ≥ 1.0	15 (100%)	47 (95.9%)	18 (100%)	0.501
High OD ratio, ≥ 3.0	8 (53.3%)	32 (65.3%)	18 (100%)	0.006

Data are expressed as the number (%) of sera. * Eighteen sera from six patients. Abbreviations: COVID-19, coronavirus disease 2019; MN, microneutralization; FIA, fluorescence immunoassay; IgG, immunoglobulin G; COI, cut-off index; ELISA, enzyme-linked immunosorbent assay; OD, optical density.

## Supplementary Materials

The following are available online at https://www.mdpi.com/2077-0383/9/7/2268/s1, Figure S1: Antibody production of asymptomatic to severe COVID-19 patients, according to the detailed severity groups; Figure S2: FIA IgM test for asymptomatic to severe COVID-19 patient; Figure S3: Antibody kinetics of two COVID-19 patients with pneumonia, serially measured by FIA IgM, FIA IgG, and ELISA total; Table S1: Clinical characteristics of asymptomatic to severe COVID-19 patient, according to the detailed severity groups; Table S2: Neutralizing antibody production and serologic tests for asymptomatic to severe COVID-19 patient, according to the detailed severity groups; Table S3: Demographics and serologic test results of negative control serum specimens.
