# Supplementary material for: Neutralizing Antibody Production in Asymptomatic and Mild COVID-19 Patients, in Comparison with Pneumonic COVID-19 Patients
Source: J Clin Med. 2020 Jul 17;9(7):2268. doi: 10.3390/jcm9072268 (PMC7408950; doi:10.3390/jcm9072268)
Supplement: Supplementary file 1 [file jcm-09-02268-s001.pdf]

Supplementary Materials

Table S1. Clinical characteristics of asymptomatic to severe COVID-19 patients, according to the detailed severity groups.

	**Asymptomatic group** *n* **= 6**	**Anosmia/dysgeusia group** *n* **= 9**	**Mild symptomatic group** *n* **= 29**	**Mild febrile group** *n* **= 20**	**Pneumonia group** *n* **= 18***
**Age**	25.2 ± 5.95	31.0 ± 8.67	29.3 ± 8.78	32.8 ± 9.64	65.7 ± 13.2
**Male sex**	4 (66.7%)	4 (44.9%)	12 (41.4%)	8 (40.0%)	3 (50%)
**Underlying disease**	0 (0%)	0 (0%)	0 (0%)	0 (0%)	6 (100%)†
**Symptom, duration**					
Anomia and/or dysgeusia	0 (0%), NA	9 (100%), 14 (5.5–21.0)	11 (37.9%), 16.0 (9.0–30.0)	5 (25.0%), 21.0 (5.5–25.5)	0 (0%), NA
Rhinorrhea and/or nasal stuffiness	0 (0%), NA	0 (0%), NA	12 (41.4%), 21.0 (14.0–49.3)	5 (25.0%), 21.0 (2.0–54.0)	0 (0%), NA
Sore throat	0 (0%), NA	0 (0%), NA	13 (44.8%), 7.0 (3.5–13.0)	5 (25.0%), 5.0 (2.5–37.0)	1 (16.7%), 5.0 (NA–NA)
Cough	0 (0%), NA	0 (0%), NA	14 (48.3%), 21.0 (8.8–39.3)	11 (55.0%), 5.0 (3.0–21.0)	4 (66.7%), 10.5 (4.0–17.8)
Sputum	0 (0%), NA	0 (0%), NA	11 (37.9%), 14.0 (7.0–30.0)	6 (30.0%), 8.0 (6.0–37.3)	1 (16.7%), 14.0 (NA–NA)
Gastrointestinal symptoms	0 (0%), NA	0 (0%), NA	7 (24.1%), 4.0 (2.0–7.0)	3 (15.0%), 7.0 (3.0–NA)	3 (50.0%), 7.0 (5–NA)
Headache	0 (0%), NA	0 (0%), NA	5 (17.2%), 2.0 (0.5–8.5)	5 (25.0%), 3.0 (1.0–5.0)	2 (33.3%), 17 (5–NA)
Fever, chill, and/or myalgia	0 (0%), NA	0 (0%), NA	0 (0%), NA	20 (100%), 3.0 (1.0–5.0)	6 (100%), 15.0 (7.3–20.8)
**Lowest Ct value**	34.0 (32.4–36.1)	33.5 (32.2–35.4)	32.2 (30.8–33.9)	32.1 (30.7–35.4)	26.4 (19.3–31.1)
**Duration of shedding**					
from symptom onset	NA	34.0 (29.5–41.5)	48.0 (32.5–45.5)	40.0 (34.0–42.0)	29.0 (23.0–38.8)
from diagnosis	32.5 (31.5–34.0)	31.0 (24.5–42.0)	33.0 (31.0–45.5)	31.5 (27.0–35.5)	23.0 (18.3–37.8)
**Sampling time, days**					
from symptom onset	NA	39.0 (34.5–46.5)	43.0 (37.0–50.0)	43.5 (37.5–47.0)	24.0 (19.8–38.3)
from diagnosis	37.0 (36.5–38.3)	35.0 (28.5–46.0)	37.0 (34.5–49.5)	35.0 (31.0–39.5)	19.5 (16.8–35.5)

Data are expressed as the number (%) of patients, mean ± standard deviation, or median (interquartile range). *A total of 18 serum samples were obtained from six pneumonic patients. Two patients with pneumonia underwent endotracheal intubation and symptoms could not be properly assessed. †Six (100%) patients had diabetes mellitus, two (33.3%) had hypertension, one (16.7%) had dyslipidemia, one (16.7%) had stable angina, one (16.7%) had gout, and one (16.7%) had thyroid cancer.

Abbreviations: COVID-19, coronavirus disease 2019; NA, not applicable; Ct, cycle threshold of RT-PCR.

Table S2. Neutralizing antibody production and serologic tests for asymptomatic to severe COVID-19 patients, according to the detailed severity groups.

	Asymptomatic group *n* = 6	Anosmia/dysgeusia group *n* = 9	Mild symptomatic group *n* = 29	Mild febrile group *n* = 20	Pneumonia group *n* = 18*
**Neutralizing antibody**					
Positive, titer ≥1:10	5 (83.3%)	7 (77.8%)	26 (89.7%)	20 (100%)	18 (100%)
High MN titer, ≥1:80	1 (16.7%)	2 (22.2%)	11 (37.9%)	7 (35.0%)	18 (100%)
**FIA, IgG**					
Positive, COI ≥1.1	6 (100%)	9 (100%)	28 (96.6%)	20 (100%)	18 (100%)
High COI value, ≥15.0	4 (66.7%)	7 (77.8%)	24 (82.8%)	20 (100%)	18 (100%)
**ELISA, total**					
Positive, OD ≥ 1.0	6 (100%)	9 (100%)	27 (93.1%)	20 (100%)	18 (100%)
High OD ratio, ≥ 3.0	3 (50.0%)	5 (55.6%)	19 (65.5%)	13 (65.5%)	18 (100%)

Data are expressed as the number (%) of sera. *Eighteen sera from six patients. Abbreviations: COVID-19, coronavirus disease 2019; MN, microneutralization; FIA, fluorescence immunoassay; IgG, immunoglobulin G; COI, cut-off index; ELISA, enzyme-linked immunosorbent assay; OD, optical density.

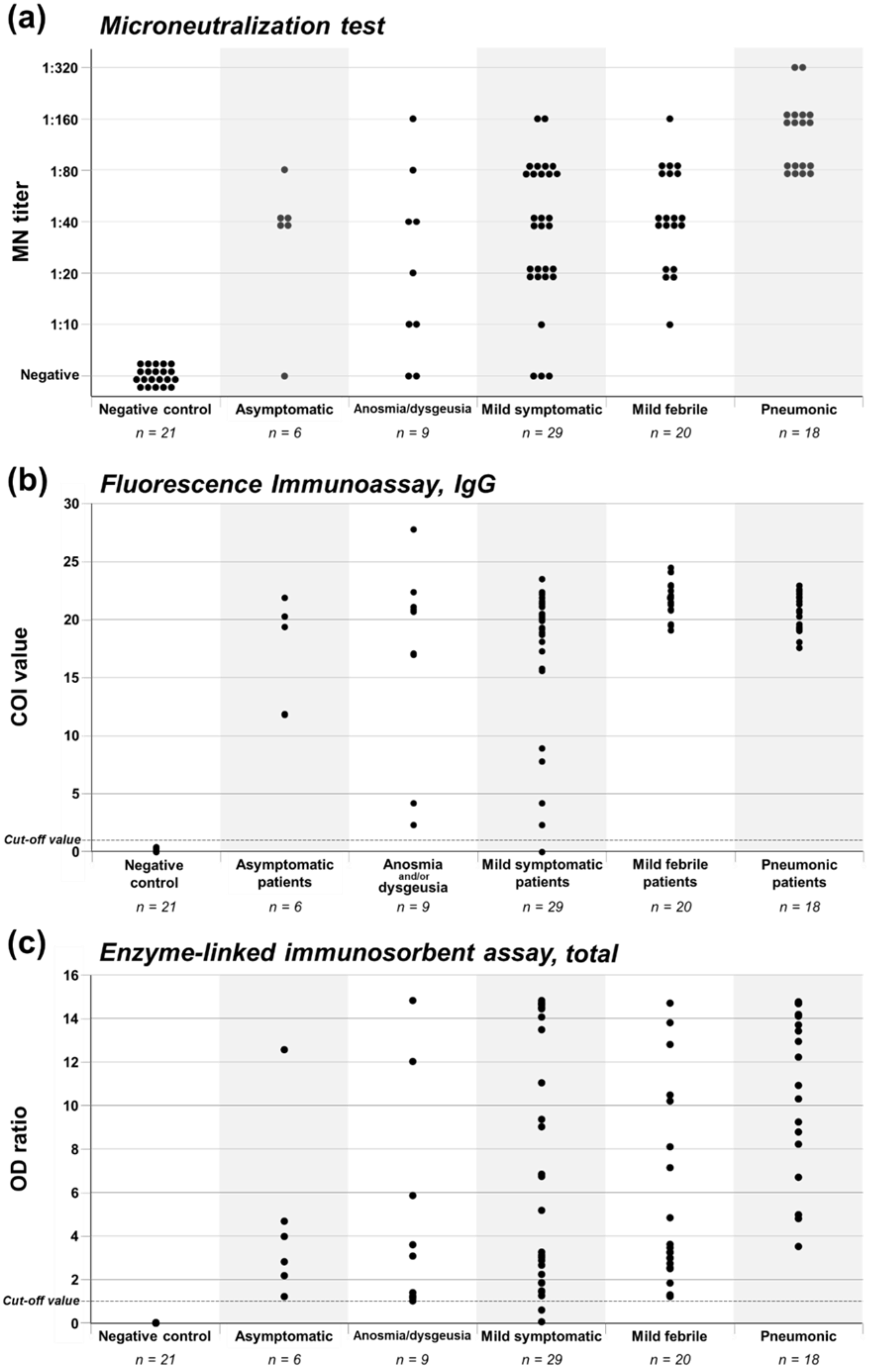

Figure S1. Antibody production of asymptomatic to severe COVID-19 patients, according to the detailed severity groups. Antibody production of asymptomatic and mild COVID-19 patients was evaluated in comparison with pneumonic COVID-19 patients. Tests were conducted by (**A**) MN test,

(**B**) FIA IgG, and (**C**) ELISA total antibody test. Abbreviations: COVID-19, coronavirus disease 2019; MN, microneutralization; FIA, fluorescence immunoassay; ELISA, enzyme-linked immunosorbent assay; IgG, immunoglobulin G; COI, cut-off index; OD, optical density.

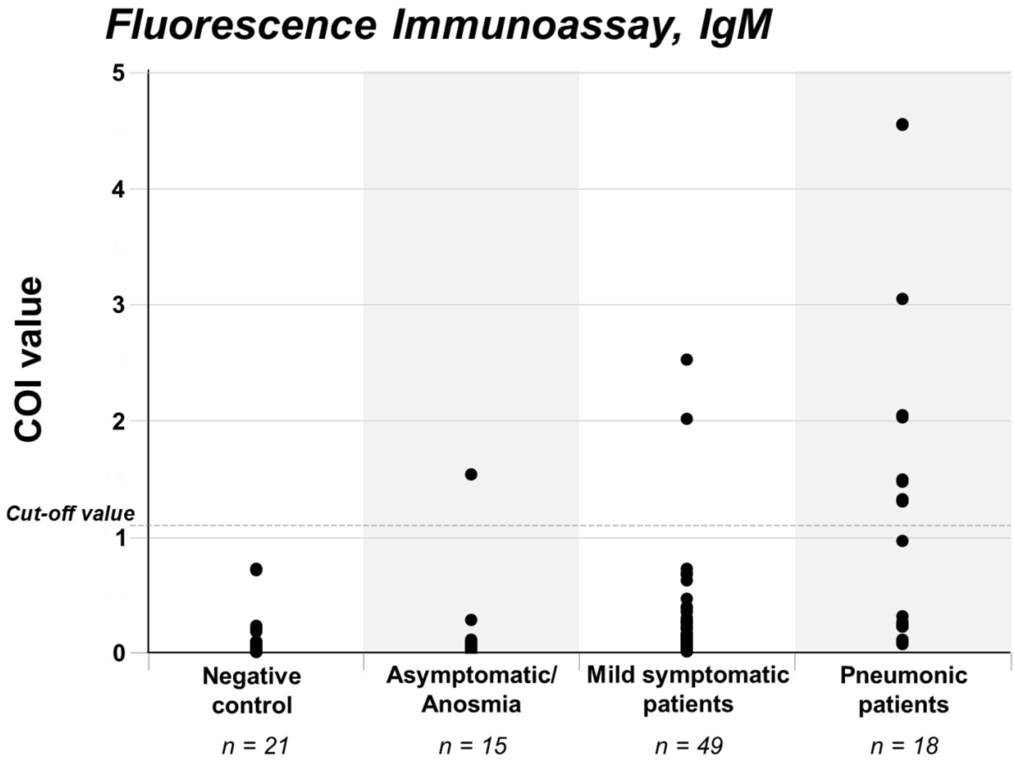

Figure S2. FIA IgM test for asymptomatic to severe COVID-19 patients. Overall, the IgM antibody measured by the IFA method was detected in three convalescent sera of asymptomatic to mild COVID-19 patients (asymptomatic to mild febrile groups; 3/64, 4.7%), while nine sera (9/18 sera, 50.0%) from three pneumonic patients (3/6 patients, 50.0%) showed positive for FIA IgM. Abbreviations: FIA, fluorescence immunoassay; IgM, immunoglobulin M; COI, cut-off index; COVID-19, coronavirus disease 2019.

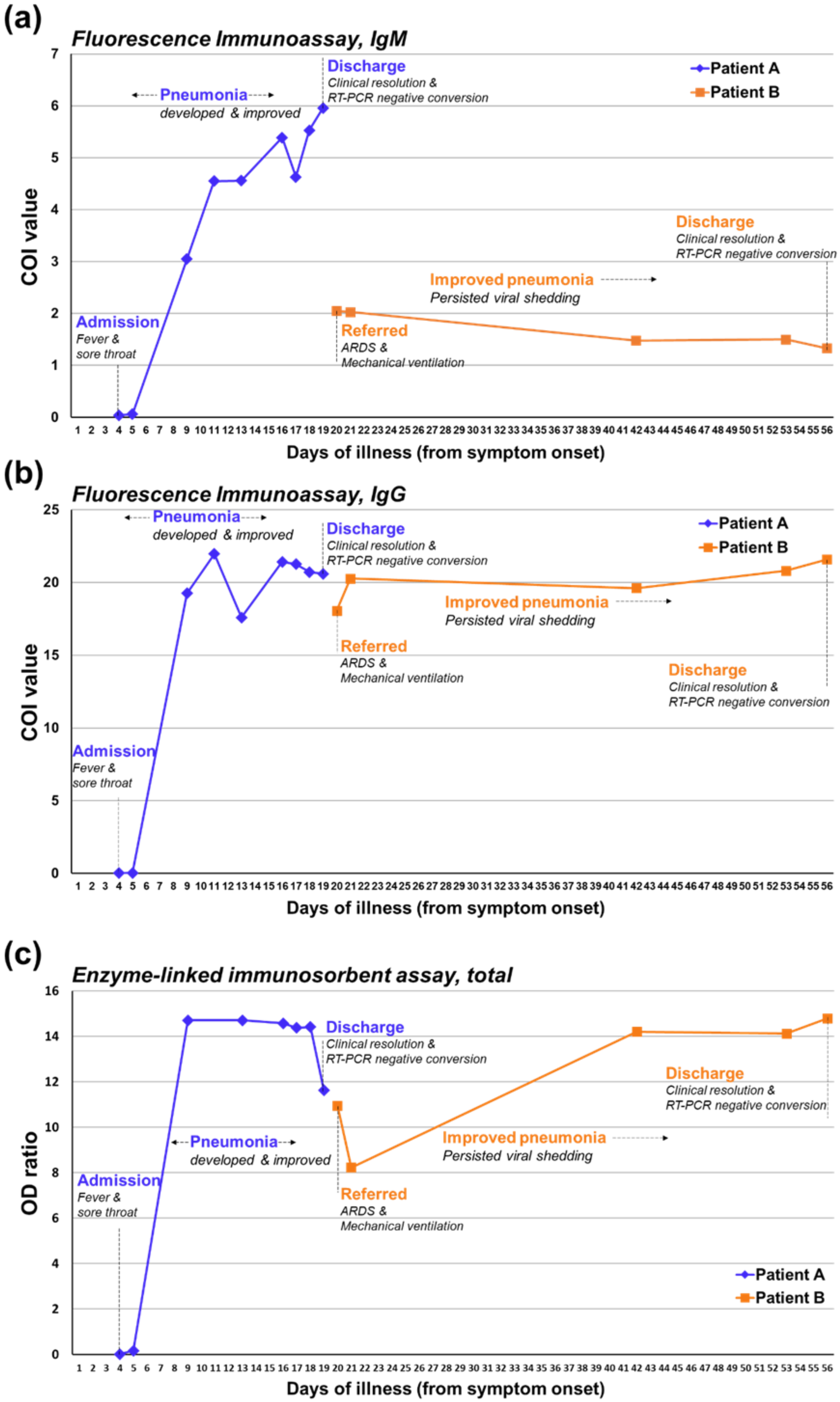
(a)
Fluorescence Immunoassay, IgM
COI value
Days of illness (from symptom onset)
Patient A
Patient B
Admission
Fever &
sore throat
Pneumonia
developed & improved
Discharge
Clinical resolution &
RT-PCR negative conversion
Referred
ARDS &
Mechanical ventilation
Improved pneumonia
Persisted viral shedding
Discharge
Clinical resolution &
RT-PCR negative conversion
(b)
Fluorescence Immunoassay, IgG
COI value
Days of illness (from symptom onset)
Patient A
Patient B
Admission
Fever &
sore throat
Pneumonia
developed & improved
Discharge
Clinical resolution &
RT-PCR negative conversion
Referred
ARDS &
Mechanical ventilation
Improved pneumonia
Persisted viral shedding
Discharge
Clinical resolution &
RT-PCR negative conversion
(c)
Enzyme-linked immunosorbent assay, total
OD ratio
Days of illness (from symptom onset)
Patient A
Patient B
Admission
Fever &
sore throat
Pneumonia
developed & improved
Discharge
Clinical resolution &
RT-PCR negative conversion
Referred
ARDS &
Mechanical ventilation
Improved pneumonia
Persisted viral shedding
Discharge
Clinical resolution &
RT-PCR negative conversion

Figure S3. Antibody kinetics of two COVID-19 patients with pneumonia, serially measured by FIA IgM (**A**), FIA IgG (**B**), and ELISA total antibody test (**C**). To present the relationship between IgM, IgG, and total antibodies against SARS-CoV-2, antibody kinetics of two COVID-19 patients with pneumonia are presented. Abbreviations: COVID-19, coronavirus disease 2019; FIA, fluorescence immunoassay; IgM, immunoglobulin M; IgG, immunoglobulin G; ELISA, enzyme-linked immunosorbent assay; COI, cut-off index; COVID-19, coronavirus disease 2019.

Table S3. Demographics and serologic test results of negative control serum specimens.

Number	Sex	Age	Respiratory virus	MN	FIA	ELISA
NC 1	M	10	Coronavirus 229E	Negative	Negative	Negative
NC 2	M	13	Coronavirus 229E	Negative	Negative	Negative
NC 3	M	53	Coronavirus 229E	Negative	Negative	Negative
NC 4	F	62	Coronavirus 229E	Negative	Negative	Negative
NC 5	M	13	Coronavirus NL63	Negative	Negative	Negative
NC 6	M	48	Coronavirus OC43	Negative	Negative	Negative
NC 7	M	11	Influenza A	Negative	Negative	Negative
NC 8	M	17	Influenza A	Negative	Negative	Negative
NC 9	F	53	Influenza A	Negative	Negative	Negative
NC 10	F	65	Influenza A	Negative	Negative	Negative
NC 11	F	7	Metapneumovirus	Negative	Negative	Negative
NC 12	M	66	Metapneumovirus	Negative	Negative	Negative
NC 13	M	16	Parainfluenza virus 2	Negative	Negative	Negative
NC 14	M	7	Respiratory syncytial virus B	Negative	Negative	Negative
NC 15	M	7	Respiratory syncytial virus B	Negative	Negative	Negative
NC 16	M	42	Respiratory syncytial virus B	Negative	Negative	Negative
NC 17	M	73	Respiratory syncytial virus B	Negative	Negative	Negative
NC 18	F	11	Rhinovirus	Negative	Negative	Negative
NC 19	F	12	Rhinovirus	Negative	Negative	Negative
NC 20	F	52	Rhinovirus	Negative	Negative	Negative
NC 21	M	67	Rhinovirus	Negative	Negative	Negative

Abbreviations: MN, microneutralization; FIA, fluorescence immunoassay; ELISA, enzyme-linked immunosorbent assay; IgG, immunoglobulin G; COI, cut-off index.
